# Religion and the natural and virtual environments: introduction

**DOI:** 10.1007/s41682-022-00105-4

**Published:** 2022-02-23

**Authors:** Julia Martínez-Ariño, Siniša Zrinščak

**Affiliations:** 1 University of Groningen, Groningen, The Netherlands; 2 Faculty of Law, University of Zagreb, Zagreb, Croatia

This special section is the outcome of the fifth biennial conference of the European Sociological Association’s Research Network 34 Sociology of Religion. It was planned to take place in Groningen, the Netherlands, on August 26–28, 2020. In light of the pandemic, we instead organised a short online event with the three keynote speakers: Giulia Evolvi (Rotterdam), Jens Köhrsen (Basel) and Pooyan Tamimi Arab (Utrecht). The first two contributed with their papers to this special section. Additionally, and despite the cancellation of the conference, Marta Kołodziejska (Warsaw), Hannah Klinkenborg and Doris Fuchs (both Münster) as well as Joram Tarusaria (Groningen) agreed to submit their full papers to this special section.

The topic of the conference was “Religion and the urban, natural and virtual environments”. It captures well three areas of religious transformation and innovation of high academic and societal relevance in contemporary European societies and beyond. With this topic, we want to address some of the main areas in which the sociology of religion has developed significantly over the last few years and where we see the potential for fruitful conversations and further theoretical, empirical and methodological development. With the notion of environments, we connect the physical, social, political and technological contexts which, in their interaction with religion, generate new social realities worth investigating. While the three environments are quite distinctive in how they interact with religion, they are also deeply entangled. The constant shaping of cities, for example, is heavily influenced by both technological innovations and climate change. Social interactions are constantly transforming in cities not only due to their size and social composition, as is underlined in previous literature, but due to the profound social transformation mainly caused by technological change and multiplication of risks. Thus, studying transformations in these three areas of life can generate theoretical and methodological insights that inform each other and the sociology of religion more broadly. The papers in the special section attest to that.

For some time, the sociology of religion has examined how urban contexts and religion are intimately linked and co-create one another. Rather than being strangers to one another, as the classical accounts of secularization theory had predicted, cities and religion, in its myriad expressions, produce new religious forms as well as new urban configurations. Some of the questions we had planned to address during the conference are the following: How do urban environments affect religious practices and, conversely, how do religious practices and groups transform urban environments? How do tourism, heritage industries and gentrification processes produce and transform urban religious heritage? Unfortunately, given the burden that the pandemic put on many of us, authors working on this strand of the conference topic had to withdraw their papers from this special section.

The second area of interest of the conference was the interaction between religion and the natural environment. Given the climate and ecological crisis we are immersed in, we consider of outmost interest to explore how religious groups and institutions position themselves with regard to this societal problem. Some questions we asked for the conference are: How do religious groups relate to the natural environment and how do they interpret the ecological crisis, and which alternatives do they suggest? Which forms does faith-based environmentalism take? How do religions position themselves in relation to other social actors in environmental concerns? While not an entirely new field of research, the study of the interaction between religion and the natural environment, as well as environmentalist movements, among other things, can still yield innovative insights that help us understand better the transformations religions are undergoing and how they in turn also influence natural environments. The articles by Hannah Klinkenborg and Doris Fuchs, Jens Köhrsen and colleagues and Joram Tarusarira in this special section contribute novel insights in this regard.

Finally, the third area of interest to us was the developments taking place in digital religion. Naturally, at the moment in which we planned the conference we could not predict the great transformations that digital religion would experience in the context of the pandemic. However, we were convinced that digital religion was an expanding field of research in the sociology of religion worth incorporating in our discussions. Many questions need further consideration, such as: How do the virtual environments and religion interact with and affect one another? How does the virtual environment influence our understandings of “religious communities”? How are religion and the concept of religion affected and challenged by the increasingly digitalized world? What conceptual, epistemological, methodological and ethical challenges are faced by scholars exploring “digital and virtual religion”? The papers by Giulia Evolvi and Marta Kołodziejska in this special section show that there is room for theoretical innovation in this area and how the pandemic has posed new challenges for researchers in the field.

In what follows, we highlight a few interconnected topics raised by the articles in in this special section.

## Religion and environmental concerns

Religious involvement in environmental issues offers a unique opportunity to understand the role of religions in society and their specific contribution to social problems in general. Still, the question remains: How do they contribute, and how can we measure that contribution? Measurement is not the crucial aspect of understanding the role of religion but examining the religious contribution leads to further unpacking of the relation between religion and the natural environment. As Jens Köhrsen, Julia Blanc and Fabian Huber (in this section) show, a premature conclusion about the “greening” of religions—which is present in the scholarship and the broader public, and which is based on some normative, declarative statements—though crucially important, misses other aspects of religious-environmental involvement. If there is a pro-ecological stand among religious groups, is it visible in all or at least in most of the religious teachings and concrete actions? Is it univocally embraced by all branches and levels of one religion, and how does this impact relations with other actors, whether religious or non-religious? While not minimising the religious involvement in environmental issues, the detailed empirical analysis of Jens Köhrsen, Julia Blanc and Fabian Huber leads to a less optimistic view. In parallel, the measurement of religious involvement on environmental issues is constrained by the great invisibility of religious actors in an extraordinary secular arena, like environmental policies. Still, as Hannah Klinkenborg and Doris Fuchs show in their study of religious actors in climate politics in the EU (in this volume), there are institutional channels for religious involvement, and invisibility should not be automatically interpreted as non-influence. There is room for religious contribution, and the question is how this operates and which implications it has for both environmental policies and religious groups.

Another relevant aspect when examining the involvement of religious groups in environmental matters is the use of “appropriate” language to be understandable to and influential for various publics, while remaining attached to the original religious background. How do religious groups solve this puzzle? The EU policy arena is an example of a secular environment that imposes the use of a secular language. Consequently, many religious organisations, not only those related to the EU, need to appropriate and employ the corresponding vocabulary. Interreligious actions that address environmental concerns may also lead to the downplaying of the specific religious background of concrete groups, thereby generating new challenges to those organisations (Köhrsen, Blanc and Huber, in this volume). Next to the use of secular language, however, and the watering down of denomination-specific elements, there are concrete religious values and deeper meanings attached to the environment and nature which need to be considered in sociological analysis. Such values are compelling as they motivate people’s actions, something which cannot be grasped if only secular language and secular reasoning are employed. If, for instance, nature is seen as an expression or a creation of God, then a pro-ecological stand has a much stronger basis (Klinkenborg and Fuchs, in this section). Respect to God as a creator involves respect to nature and other humans, and this—whether expressed in more religious or in more secular terms—is a specific religious contribution. In addition, not respecting such values may cause serious social conflicts. If people perceive the land as sacred, then pure economic or legal reasoning is not of much help when understanding and addressing conflicts related to climate change (Tarusarira, in this section). What may often be superficially perceived as only an economic or legal conflict can have moral underpinnings. Therefore, a conflict may also emanate from contrasting values or cultural aspects that are frequently ignored by policymakers and academics alike. Consequently, if such moral elements are considered, rational policy processes will not neglect religious meanings as non-rational or outdated but will consider them to understand people’s values and their impact on social processes. There are also other values, such as global and intergenerational justice, which are essential when considering climate change and the environment. However, these are often overlooked or dismissed by a variety of actors, and, as research has shown, religious organisations may be the advocates of those values (Klinkenborg and Fuchs, in this section).

The diffusion and translation of religious norms and ideas into broader contexts is hence a complex two-way process. It is a process in which religious groups affect the wider society and one in which the social perception of the influence that religions can have on environmental discussions affects back religions and their ways of dealing with such discussions. The first side of the process, namely the religious contribution to society, may, as the papers in this special section confirm, involve conflict and can be perceived by other non-religious actors as conflictual. While conflict dynamics are not the only way to look at the role of religion in society, it is an important aspect worth considering. Tensions and conflicts appear between religious and secular actors, but intradenominational, interdenominational and interreligious tensions and conflicts also exist (Klinkenborg and Fuchs, in this section; Köhrsen, Blanc and Huber, in this section). All these factors, in turn, affect religions and may change the ways they engage in environmental discussions and activism, which is the second side of the process we referred to above.

## Space and movement in the (non)digital era

Environmental issues and conflicts, and the development of new digital technologies change the relationship of humans to (social) space. The climate crisis is generating, and will generate even more, the displacement of people from their places of origin and residence. Attachment to those places may become more volatile or vulnerable, especially in those regions of the world where vulnerabilities to the effects of climate change are more pressing. The attachment to a place, though, needs to be examined in all its dimensions to fully grasp how it operates. The case when people do not want to move, as examined in Joram Tarusarira’s piece, stresses a deep meaning of belonging to a particular place that is linked to the sacred character assigned to territory. This perspective complements the work on involuntary migration and issues of negotiating identity in new social contexts. Concepts of “emplacement” and “disemplacement”, as discussed in the article by Joram Tarusarira, may be of help by illustrating that people do not want to be displaced from the context to which they are deeply attached. Perception and feeling of land as a sacred value is an innovative angle developed in the article that can be replicated in other studies focusing, for instance, on values and culture as well as analysis of globalisation and resistance to globalisation. Observing and listening in local contexts is a vital part of analysing broader social processes.

Similarly, living in the so-called digital world has profoundly changed our relation to space, movement and the material world. Is the digital becoming a new reality with its power to radically transform the sense of what is material, what is present and what is touchable? Consequently, can we consider “digital religion” a new way of being religious connected to new modes of sensing and being religious? The digital world is an entirely new and transformative reality, but it is not wholly distinct from the non-digital world (Evolvi, in this section). Digital technologies change our lives, but there are no clear boundaries between the “online” and the “offline” worlds, and they both constitute the social reality. The blurriness of such distinction is similar to other boundaries we usually research, like those between the religious and the secular or between the public and the private. However, a combination of both “online” and “offline” worlds may well be a starting point for the analysis. In the case of religion, online possibilities are interlinked with a core element of religion, which is human bodily existence (Evolvi, in this section). From Émile Durkheim and William James, classics in studying religion, to many contemporary social scientists, we have learnt that the very nature of religion is connected to the collective and material dimensions of human life. The question is then how do digital technologies help, obviously in transformative ways, to sense religion when co-presence of bodies and materiality are limited? Similarly, the digitalisation of life transforms space. Therefore, we need to reconceptualise it in order to capture various types of spaces that are generated and the new ways in which people experience them in their religious engagements (Evolvi, in this section). The ongoing pandemic illustrates well how religions use the digital possibilities available to continue their core missions and how people satisfy their religious needs by engaging digitally (Evolvi, in this section; Kołodziejska, in this section). The spatial-temporal continuity that digital technologies have offered during the abrupt rupture provoked by the pandemic does not imply that human communication, and religious activities are part of that, has not changed profoundly. On the contrary, as the study of digital religion illustrates, digital technologies have generated entirely new forms of encounter and communication which have facilitated a sense of continuity.

## Religion, the environment and digital worlds: power and transformation

As already underlined, topics of religion, the natural environment and digital worlds are interlinked in many ways. They offer a fertile ground for studying (partly) new issues, though with the pressing need to rethink existing knowledge. From the five articles that form this special section, many issues appear as relevant. Here we highlight two of them that we understand as the core of sociological analysis of social processes: power structures and social change.

Power structures and social inequalities are lenses often employed to understand society. Their importance in understanding religion is also well documented in this selection of articles. Are religions powerful in political and social arenas, and in which ways? In which societal spheres are they powerful, and in which ones not and why? Environmental involvement of religions in the EU policy process (Klinkenborg and Fuchs, in this section) and the issue of the “greening” of religions (Köhrsen, Blanc and Huber, in this section) demonstrate the need to analyse the role of religions inside existing power structures. Do religious organisations contribute to the change of existing power structures or do they rather resist change and favour the *status quo*? Examples of the need to consider power structures abound in the articles. As Joram Tarusarira shows in his analysis of a land conflict in Zimbabwe, economic and political interests prevailed over local people’s attachment to land and nature, which was neglected. This was mediated by the unequal power position of various actors. Adopting a critical perspective that takes power into consideration also points to inequalities between religions. In their analysis of faith-based contributions to the EU policy discourse on climate change, Hannah Klinkenborg and Doris Fuchs show that not all religious groups enjoy the same possibilities of acting in the secular policy arena, and tensions between and among religions are partly connected to their overall positions in society (Köhrsen, Blanc and Huber, in this section). The disadvantaged social position of one’s religion, as the Polish case analysed by Marta Kołodziejska demonstrates, is not only conditioned by being a real minority but the minority status is further strengthened by the privileged position occupied by the dominant groups, in this case the Roman Catholic Church. Hence, in this particular analysis, the same seemingly neutral government provision regarding the COVID-19 pandemic results in the deepening of existing inequalities between religious groups.

Issues of power and inequalities are also crucial for understanding social change in the digital arena. Studying digital religion and analysing how the digital world transforms the way of being religious is conditioned by a very first question: who has access to the digital world? A romantic view of broad and democratic access to digital technologies for everyone and everywhere is inaccurate and does not help in studying digital religion. There are at least three types of inequalities related to digital access. The first one is socio-economic, with significant age, gender, and ethnic dimensions. Access to digital technologies is conditioned by economic means and by the knowledge of using new technologies. If religions go online, this influences who and in which way will be able to join (Kołodziejska, in this section). The second type of inequality emerges between religions, as some are economically and politically better positioned to use modern technologies and combine online and offline settings to respond to unexpected situations like the lockdown (Kołodziejska, in this section). The third sort of inequalities refers to the global dimension of digital access. Giulia Evolvi underlines in her work that scholarship on digital religion is mainly based on the North American and European contexts, which hardly ever reflects the situation in other parts of the world. Therefore, we lack knowledge of how digital religion develops in non-Western contexts.

Using the angle of power and inequalities does not mean that structures are petrified and there are no changes. On the contrary, the articles in this special section demonstrate the power of religions to be transformative and innovative. Protecting the natural environment as a pressing social issue illustrates this, as it generates new ways of acting, speaking and rethinking religious teachings. This sheds a different light on the innovation processes taking place within religions and their role in societal transformation processes (Köhrsen, Blanc and Huber, in this section). The tensions among and between religions and societies mentioned above are part of these innovation processes. By choosing a firm attitude toward environmental issues, religious organisations promote changes and call for concrete actions (Klinkenborg and Fuchs, in this section). Also, the ongoing pandemic has posed an ultimate need for changing our usual patterns of living. As other groups in society, religions reacted in several innovative ways, not only by using online possibilities. Adaptation to challenges is an ongoing reality for all religions. Even more, religions are part of a profound social transformation that questions existing ways of thinking about space and materiality (Evolvi, in this section) and how we use and understand communication (Kołodziejska, in this section). Taking into account religious values transforms the relations between key stakeholders in one’s society, which is essential for scientific analysis (Tarusarira, in this section).

The papers in this special section shed light on a variety of developments where religious groups, practices, values, ideas and beliefs play crucial roles. With their agency, religious groups transform the environments of which they are a part and, in turn, are transformed by those environments. Scholarship in the sociology of religion will have to continue paying attention to these challenges, innovations and tensions that religious and other social groups face in a time of socio-political and environmental instability, which is additionally aggravated by the health and care crisis brought up by the COVID-19 pandemic.

